# Comparative transcriptome analysis reveals whole-genome duplications and gene selection patterns in cultivated and wild *Chrysanthemum* species

**DOI:** 10.1007/s11103-017-0663-z

**Published:** 2017-10-19

**Authors:** So Youn Won, Soo-Jin Kwon, Tae-Ho Lee, Jae-A Jung, Jung Sun Kim, Sang-Ho Kang, Seong-Han Sohn

**Affiliations:** 1 Genomics Division, National Institute of Agricultural Sciences, Rural Development Administration, Jeonju, 54874 Republic of Korea; 2 Research Policy Bureau, Rural Development Administration, Jeonju, 54874 Republic of Korea; 3 Floriculture Research Division, National Institute of Horticultural and Herbal Science, Rural Development Administration, Wanju, 55365 Republic of Korea

**Keywords:** Whole-genome duplication, Transcriptome, Asteraceae, *Chrysanthemum morifolium*, *Chrysanthemum boreale*

## Abstract

**Key message:**

Comparative transcriptome analysis of wild and cultivated chrysanthemums provides valuable genomic resources and helps uncover common and divergent patterns of genome and gene evolution in these species.

**Abstract:**

Plants are unique in that they employ polyploidy (or whole-genome duplication, WGD) as a key process for speciation and evolution. The *Chrysanthemum* genus is closely associated with hybridization and polyploidization, with *Chrysanthemum* species exhibiting diverse ploidy levels. The commercially important species, *C. morifolium* is an allohexaploid plant that is thought to have originated via the hybridization of several *Chrysanthemum* species, but the genomic and molecular evolutionary mechanisms remain poorly understood. In the present study, we sequenced and compared the transcriptomes of *C. morifolium* and the wild Korean diploid species, *C. boreale*. De novo transcriptome assembly revealed 11,318 genes in *C. morifolium* and 10,961 genes in *C. boreale*, whose functions were annotated by homology searches. An analysis of synonymous substitution rates (Ks) of paralogous and orthologous genes suggested that the two *Chrysanthemum* species commonly experienced the Asteraceae paleopolyploidization and recent genome duplication or triplication before the divergence of these species. Intriguingly, *C. boreale* probably underwent rapid diploidization, with a reduction in chromosome number, whereas *C. morifolium* maintained the original chromosome number. Analysis of the ratios of non-synonymous to synonymous nucleotide substitutions (Ka/Ks) between orthologous gene pairs indicated that 107 genes experienced positive selection, which may have been crucial for the adaptation, domestication, and speciation of *Chrysanthemum*.

**Electronic supplementary material:**

The online version of this article (doi:10.1007/s11103-017-0663-z) contains supplementary material, which is available to authorized users.

## Introduction

The genus *Chrysanthemum* shows considerable diversity in terms of ploidy level, as well as flower shape, color, and size. This genus comprises approximately 40 species that exhibit various degrees of polyploidy, from diploid to decaploid, with nine chromosomes as the basal unit (Dowrick 1952; Liu et al. 2012). The commercial species, *C. morifolium*, is used as an ornamental and medicinal plant worldwide and appears to be a hexaploid species (2n = 6x = 54). Cytogenetic, molecular phylogenetic, and genetic studies suggested that *C. morifolium* originated from the natural hybridization of species such as *C. indicum, C. lavandulifolium, C. nankingense, C. vestitum*, and *C. zawadskii* (Chen 1985; Dai et al. 1998, 2005; Ma et al. 2016; Zhou and Silan 2001). However, the extent and timing of evolutionary events such as polyploidy or whole-genome duplication (WGD) in *C. morifolium* remain uncertain.

WGD is one of the key factors in the evolution, speciation, and diversification of the angiosperm lineage (Soltis et al. 2009; Soltis and Soltis 2016; Van de Peer et al. 2009b; Wendel et al. 2016). Genome and transcriptome sequence analyses have revealed that angiosperms shared an ancient WGD known as paleopolyploidy and additional recent lineage-specific WGDs (Jiao et al. 2011; Tang et al. 2008; Van de Peer et al. 2009a). After WGD, gene pairs that were duplicated within species (also known as paralogs) are retained or lost in the mode of neo-, sub-, or non-functionalization followed by extensive genome rearrangement and diploidization (Panchy et al. 2016; Soltis et al. 2015; Van de Peer et al. 2009b). Such post-WGD events are variable and extensive enough to result in reproductive isolation and ultimately, speciation (Paterson et al. 2010; Vanneste et al. 2014). The cycles of WGD and the subsequent dynamic changes are recurrent events that are associated with the radiation and diversity of angiosperm species (Paterson et al. 2010; Soltis and Soltis 2016; Vanneste et al. 2014).

With the accumulation of genome-wide nucleotide sequences, evolutionary events such as WGD and speciation can be successfully inferred by evaluating the age distribution of homologous gene pairs (Blanc and Wolfe 2004; Van de Peer et al. 2009a). Synonymous nucleotide substitutions of protein-coding genes are not accompanied by amino acid changes, and are thus neutral and free from natural selection (Blanc and Wolfe 2004). Therefore, the rate of synonymous substitutions (Ks) is proportional to the time lapse since the generation of two homologous genes and is thus used to approximate the timing of the occurrence of homologs, much like a molecular clock (Blanc and Wolfe 2004). Because WGD results in the production of excessive paralogous gene pairs of a particular age, the Ks distribution of paralogs displays a peak of high density at a specific Ks value from which the timing of WGD is deduced (Blanc and Wolfe 2004). Similarly, the Ks distribution of homologous gene pairs between two different species (also known as orthologs) is used to determine the time of speciation (Blanc and Wolfe 2004). The rate of nonsynonymous substitutions (Ka) and the Ka/Ks ratio also serve as useful parameters to investigate the molecular evolution of two species that have diverged (Fay and Wu 2003). Because synonymous substitution occurs more frequently than nonsynonymous substitution, the Ka/Ks ratios of most orthologous genes are less than one, indicating that the gene pair is under purifying/negative selection (Fay and Wu 2003). By contrast, orthologs under adaptive/positive selection exhibit Ka/Ks values greater than one, providing insights into the molecular evolutionary framework underlying adaptation, divergence, and speciation (Fay and Wu 2003).

The Asteraceae (Compositae), one of the largest and most diverse plant families, includes 13 subfamilies, 45 tribes, 1,911 genera, and 32,913 species (Fu et al. 2016; MBG 2013). The phylogenetic relationships among the major lineages of Asteraceae have been thoroughly investigated via comparisons of genes in the chloroplast or nucleus (Linder et al. 2000; Panero et al. 2014), and information about genome evolution within Asteraceae was also recently obtained. A comparison of linkage maps between *Lactuca sativa* and *Vitis vinifera* revealed the occurrence of paleohexaploidy in Asteraceae (Truco et al. 2013), which was confirmed in four major subfamilies (Asteroideae, Cichorioideae, Carduoideae, and Mutisiodeae) by analyzing the Ks distributions for 18 species (Barker et al. 2008). Additionally, Asteroideae and Mutisiodeae experienced another round of WGD (Barker et al. 2008). These lineage-specific WGDs were also detected in several tribes or genera showing species richness (Huang et al. 2016; Panero and Crozier 2016). Similar analyses using more diverse taxa showed that there were two rounds of WGD, one prior to the divergence between Asteraceae and its sister family Calyceraceae and another specific for the core Asteraceae (Barker et al. 2016; Huang et al. 2016). Although these studies have characterized the evolutionary history in representative lineages of Asteraceae, few studies have been conducted on the *Chrysanthemum* genus on a genome-wide scale.

In the present study, we sequenced the transcriptome of *C. morifolium* and subjected it to molecular evolutionary analysis. For comparison, we included the diploid wild species, *C. boreale* (2n = 2x = 18), which is native to Korea. We inferred WGD and species divergence time by identifying the paralogs and orthologs within and between species and plotting their Ks distribution. Finally, we identified genes under positive selection, providing genome-wide information about genes that may have been involved in the domestication or adaption of *C. morifolium*.

## Materials and methods

### Plant materials

*C. morifolium* (cv. Baekma) plants were kindly provided by the National Institute of Horticultural and Herbal Science (NIHHS), Republic of Korea. *C. boreale* (IT121002) was collected from the Republic of Korea as previously described (Hwang et al. 2013). All plants were propagated by stem cuttings and grown in a greenhouse in NIHHS under natural light conditions.

### RNA extraction, library construction, and sequencing

Total RNA was extracted from leaf tissue using TRIzol Reagent (Invitrogen, USA). RNA quantity and quality were evaluated using a NanoDrop spectrophotometer (NanoDrop Technologies, USA), electrophoresis on a 1% denaturing agarose gel, and an Agilent 2100 Bioanalyzer (Agilent Technologies, USA). GS FLX cDNA library construction, emulsion PCR, and pyrosequencing were conducted at the National Instrumentation Center for Environment Management (NICEM, Seoul National University, Republic of Korea; http://nicem.snu.ac.kr) following standard procedures. Approximately 5 µg of total RNA per sample was used for library construction, as described in the cDNA Rapid Library Preparation Method Manual provided with GS FLX Titanium Series reagents (Roche, USA). The libraries were amplified using emPCR kits (Roche, USA) and sequenced on a 454 GS FLX Titanium Sequencer (Roche, USA) according to the manufacturer’s instructions. The sequencing data were deposited in the National Center for Biotechnology Information (NCBI) Sequence Read Archive with accession numbers SRR5768981 and SRR5768982.

### Assembly and functional annotation of the transcriptome

The raw data were assembled using Newbler ver. 2.6 software (Roche, USA, http://www.454.com) with the cDNA option for transcriptome assembly. Trimming and assembly of raw reads resulted in the production of isotigs or contigs (equivalent to transcripts, including splice variants), which were grouped into isogroups (equivalent to genes). Additionally, raw reads that were not assembled into isotigs or contigs were remained as singletons and preprocessed using the Lucy DNA sequence quality and vector trimming tool (Chou and Holmes 2001). After removing short singletons less than 200 bp, redundant singletons were filtered out using the CD-HIT-EST program with the option −c 0.90 (90% sequence identity) −n 10 −r 1 (comparing both strands) (Li and Godzik 2006).

The assembled transcripts and singletons were annotated by homology searches using the Basic Local Alignment Search Tool (BLASTX) against the UniProtKB/Swiss-Prot database (http://web.expasy.org/docs/swiss-prot_guideline.html) and the NCBI Viridiplantae (green plants) non-redundant (nr) protein database with an e-value threshold of 1e-5. Based on annotation using the Swiss-Prot database, Gene Ontology (GO) terms were assigned using the BLAST2GO program (Conesa et al. 2005). For each GO term, the p-value for the number of genes between the two species was calculated via Pearson’s Chi-Square test using the WEGO program (Ye et al. 2006). The GO distribution at level 2 was plotted.

### Identification of putative orthologs and paralogs

Proteins and transcripts sequences for globe artichoke (*Cynara cardunculus* var. *scolymus*) were downloaded from the Globe Artichoke Genome Database (http://www.artichokegenome.unito.it). Open reading frames (ORFs) for *Chrysanthemum* species were determined using TransDecoder (Haas et al. 2013) with the option minimum ORF size of 300 bp and minimum protein length of 50 amino acids. For transcripts resulting in multiple ORFs, the longest ORF was used for analysis. Since redundancy via alternative splicing also biases downstream analysis, the longest transcript per isogroup or unigene was subjected to analysis. Orthologs and paralogs among the three species were identified by conducting OrthoMCL analysis (Li et al. 2003). Briefly, all-against-all BLASTP searches were performed using the translated protein sequences with an e-value threshold of 1e-10 and an identity threshold of 50%, and the Markov Clustering Algorithm was used to cluster similar sequences into orthologous and paralogous groups. Orthologous gene pairs were retrieved from orthogroups containing one gene per species. For each species, paralogous genes were determined from orthogroups containing two genes per species, regardless of the contribution of the other species.

### Estimation of divergence time, and detection of WGD and positively selected genes

The analysis was conducted as previously described (Blanc and Wolfe 2004; Kim et al. 2014). For each ortholog or paralog, the protein sequences were aligned using ClustalW (Larkin et al. 2007) and the corresponding codons were aligned using PAL2NAL (Suyama et al. 2006) with the guidance of coding sequences (CDSs). The Ka and Ks values were calculated using the Nei–Gojobori method (Nei and Gojobori 1986) implemented in the PAML package and subjected to Fisher’s exact test to determine p-values (Yang 1997). The Ks distributions were fitted with log-Gaussian mixture models using Gaussian Mixture Models with Bayes Factors and plotted. The time of WGD and species divergence occurrence was calculated using the following equation: T = Ks/(2 × 1.5 × 10^−8^ substitutions/synonymous site/year) (Koch et al. 2000). To classify genes according to the selection type, the Ka/Ks score was calculated for each orthologous gene pair. Orthologous gene pairs with Ks > 0.1 were excluded from analysis to identify genes under positive selection to avoid the identification of potential paralogs (Cheng et al. 2015).

## Results

### Sequencing and assembly of transcriptomes

**Fig. 1:**
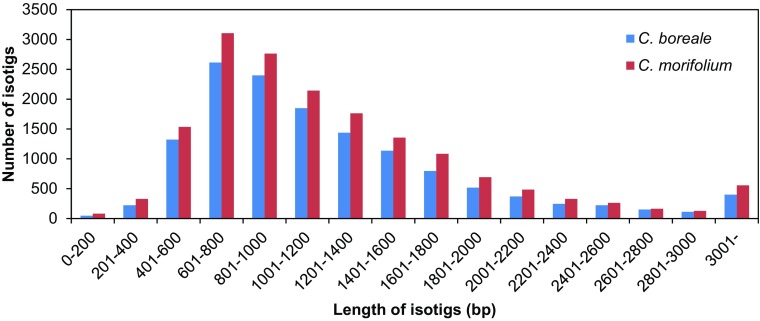
Length distribution of de novo assembled transcripts. *C. boreale* and *C. morifolium* transcripts are represented by blue and red bars, respectively

The pyrosequencing of cDNA libraries yielded 673,206 and 660,419 raw reads for *C. boreale* and *C. morifolium*, respectively, with an average length of 445 bp in both libraries (Table 1). De novo assembly of preprocessed reads resulted in the identification of 10,961 isogroups containing 13,841 isotigs for *C. boreale* and 11,318 isogroups containing 16,769 isotigs for *C. morifolium* (Table 1). Isotigs from the two species showed similar length distributions, with an average length of 1216 bp for *C. boreale* and 1230 bp for *C. morifolium*, although the number of obtained isotigs was greater in *C. morifolium* than in *C. boreale* (Fig. 1; Table 1). Additionally, 76,428 and 97,947 raw reads were retained as singletons and further preprocessed into 41,368 and 57,035 clean reads for *C. boreale* and *C. morifolium*, respectively, with an average length of ~ 480 bp (Table 1).

**Table 1:** Summary of transcriptome pyrosequencing and assembly in two *Chrysanthemum* species

Parameters	*C. boreale*	*C. morifolium*
(A) Pyrosequencing		
Number of raw reads	673,206	660,419
Total length of raw reads (Mbp)	300	294
Mean length of raw reads (min–max)	445 (40–1027)	445 (40–1203)
(B) Assembly		
Number of isogroups (genes)	10,961	11,318
Number of isotigs (transcripts)	13,841	16,769
Mean length of isotigs (min–max)	1216 (62–11,544)	1230 (62–11,544)
Number of singletons	76,428	97,947
Number of singletons after preprocessing	41,368	57,035
Mean length of singletons after preprocessing (min–max)	480 (200–940)	482 (200–1023)

### Functional annotation of transcripts

The transcripts were annotated based on sequence homology searches. First, BLASTX analysis against Viridiplantae proteins in the NCBI nr database revealed that 10,426 (95.12%) and 10,591 (93.58%) isogroups had significant matches in *C. boreale* and *C. morifolium*, respectively (Table 2). In both species, the species with the most BLASTX hits was *Cynara cardunculus* var. *scolymus*, followed by *Vitis vinifera, Sesamum indicum*, and *Coffea canephora*. A comparison to the UniProtKB/Swiss-Prot protein database showed that 8957 (81.72%) and 9013 (79.63%) isogroups returned positive hits in *C. boreale* and *C. morifolium*, respectively (Table 2). Overall, more than 90% of isogroups were functionally annotated by at least one public protein sequence database, which was also observed for isotigs (Table 2). However, the percentage of sequences that had significant BLAST hits was lower in singletons than in isogroups and isotigs. A total of 27,196 (65.74%) and 34,296 (60.13%) singletons were annotated in *C. boreale* and *C. morifolium*, respectively (Table 2). Similar to the isogroups, *Cynara cardunculus* var. *scolymus, V. vinifera*, and *Coffea canephora* were the top three species that were mostly highly represented in BLAST.

**Table 2:** Summary of functional annotation of the transcriptomes of two *Chrysanthemum* species

	*C. boreale*		*C. morifolium*	
(A) Isogroups				
BLAST match (Viridiplantae)	10,426	(95.12%)	10,591	(93.58%)
BLAST match (Swiss-Prot)	8,957	(81.72%)	9,013	(79.63%)
BLAST match	10,442	(95.27%)	10,594	(93.60%)
GO annotations	8,380	(76.45%)	8,445	(74.62%)
(B) Isotigs				
BLAST match (Viridiplantae)	12,915	(93.31%)	15,523	(92.57%)
BLAST match (Swiss-Prot)	11,079	(80.04%)	13,355	(79.64%)
BLAST match	12,932	(93.43%)	15,526	(92.59%)
GO annotations	10,420	(75.28%)	12,657	(75.48%)
(C) Singletons				
BLAST match (Viridiplantae)	27,014	(65.30%)	34,112	(59.81%)
BLAST match (Swiss-Prot)	19,045	(46.04%)	23,918	(41.94%)
BLAST match	27,196	(65.74%)	34,296	(60.13%)
GO annotations	16,995	(41.08%)	23,918	(41.94%)

The percentages of functionally annotated genes are shown in parentheses

**Fig. 2:**
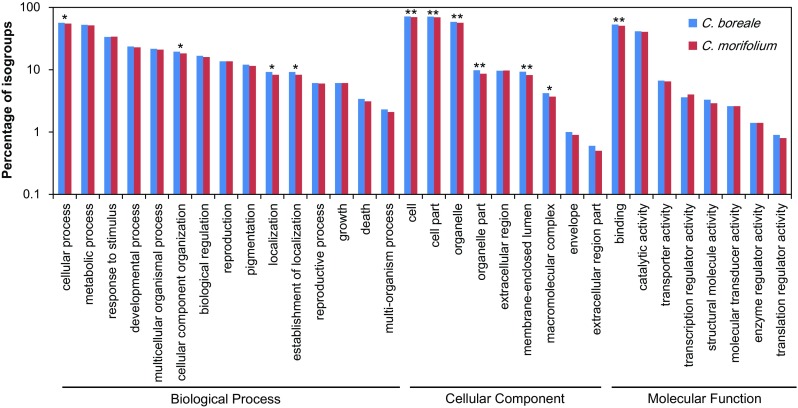
Gene ontology (GO) classification of isogroups. Annotation results from Swiss-Prot were mapped to the second level of GO terms, which are represented by blue and red bars for *C. boreale* and *C. morifolium*, respectively. Statistically significant differences between the two species are indicated (**p < 0.01 and *p < 0.05)

To categorize the functions of the transcripts, we assigned them to Gene Ontology (GO) terms based on Swiss-Prot annotation. In general, the GO distributions were highly similar in both species and included various categories, although several GO terms were significantly different between the two species (Fig. 2). In *C. boreale*, a total of 8380 (76.45%) isogroups were successfully mapped to one or more GO terms (Table 2), with 7902 (72.09%) assigned to biological process, 8259 (75.35%) to cellular component, and 7841 (71.54%) to molecular function. Likewise, in *C. morifolium*, 8445 (74.62%) isogroups were assigned to GO terms (Table 2), including 7919 (69.97%) to biological process, 8304 (73.37%) to cellular component, and 7888 (69.69%) to molecular function. In contrast to the isogroups, the annotation rates were lower for singletons, with 16,995 (41.08%) *C. boreale* and 23,918 (41.94%) *C. morifolium* transcripts mapped to GO terms (Table 2). The singletons included genes with relatively low expression levels and many were functionally annotated; however, since the singletons were very short compared to isogroups (Table 1), it is possible that they also included contaminants or artifacts derived from cDNA synthesis and sequencing (Meyer et al. 2009). Therefore, singletons were excluded from subsequent analyses.

### Clustering of transcripts

**Fig. 3:**
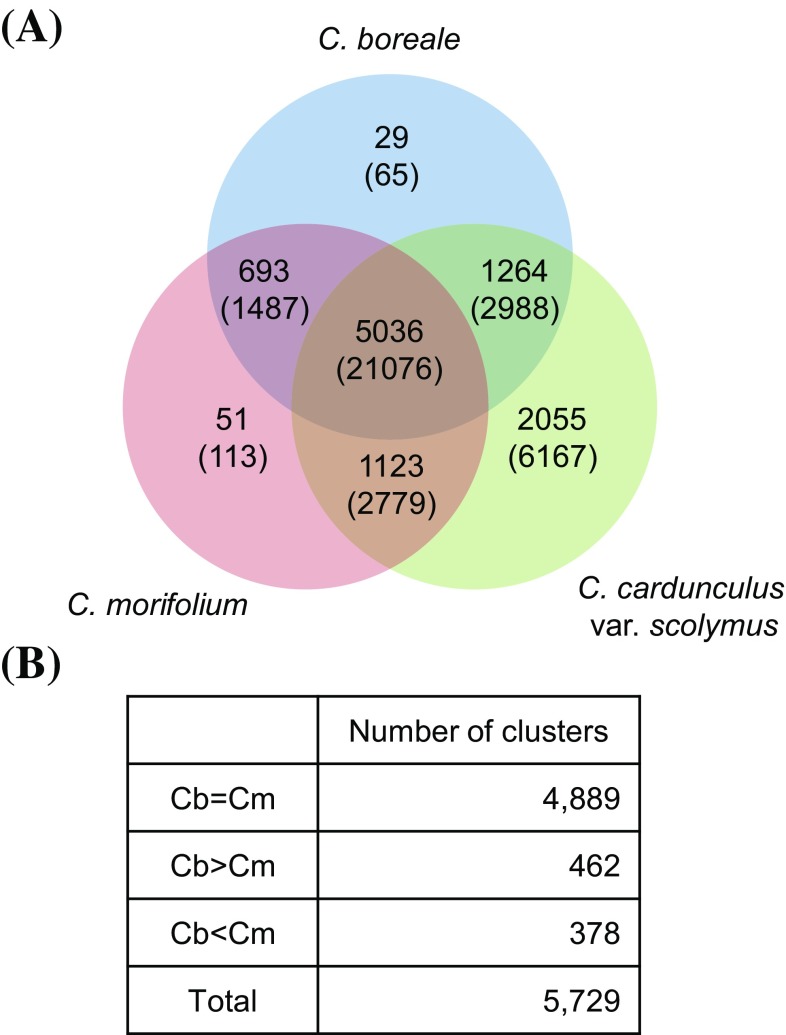
Summary of OrthoMCL analysis. **a** Venn diagram showing OrthoMCL-based gene clusters in *C. boreale, C. morifolium*, and *Cynara cardunculus* var. *scolymus*. The number of gene clusters and genes (within parenthesis) is indicated for each section. **b** Classification of *Chrysanthemum* orthoclusters based on the contribution of each species

To compare the transcriptome profiles of the two species, we constructed orthologous gene clusters using OrthoMCL. Among the species whose genome sequences were available, we included the phylogenetically closest species, *Cynara cardunculus* var. *scolymus*, to investigate evolutionary events (see below). A total of 5036 orthologous clusters consisting of 21,076 genes were common to all three species (Fig. 3), 2970 of which were identified as single-copy gene clusters and used to estimate the divergence time of species. We counted 29, 51, and 2055 species-specific orthogroups in *C. boreale, C. morifolium*, and *Cynara cardunculus* var. *scolymus*, respectively (Fig. 3). Comparison of the two genera revealed that 773 and 2055 gene clusters were unique to *Chrysanthemum* and *Cynara*, respectively (Fig. 3).

The two *Chrysanthemum* species shared 5729 gene clusters, whereas 1293 and 1174 gene families were specific to *C. boreale* and *C. morifolium*, respectively (Fig. 3a). We compared the number of genes per orthologous cluster in the 5729 clusters of the two species and found that 4,889 clusters contained the same number of genes in both species (Fig. 3b); in particular, 4512 were identified as single-copy orthogroups. However, 462 and 378 clusters contained more genes from *C. boreale* and *C. morifolium*, respectively, than from the other species (Fig. 3b). Specifically, clusters with a 2:1 gene ratio in *C. boreale* and *C. morifolium* were the most abundant, accounting for 325 groups, followed by the opposite (1:2) ratio for 276 groups. When we included species-specific clusters without the contribution of the other *Chrysanthemum* counterpart, we obtained a similar result, as 1755 and 1552 orthologous groups had surplus genes from *C. boreale* and *C. morifolium*, respectively.

In addition to orthologs, clustering analysis revealed 978, 945, and 4,326 paralogous gene clusters for *C. boreale, C. morifolium*, and *Cynara cardunculus* var. *scolymus*, respectively. Among these, 731, 719, and 2715 clusters included pairs of paralogous genes, which was used to examine WGD in each species.

### Species divergence time and WGD

**Fig. 4:**
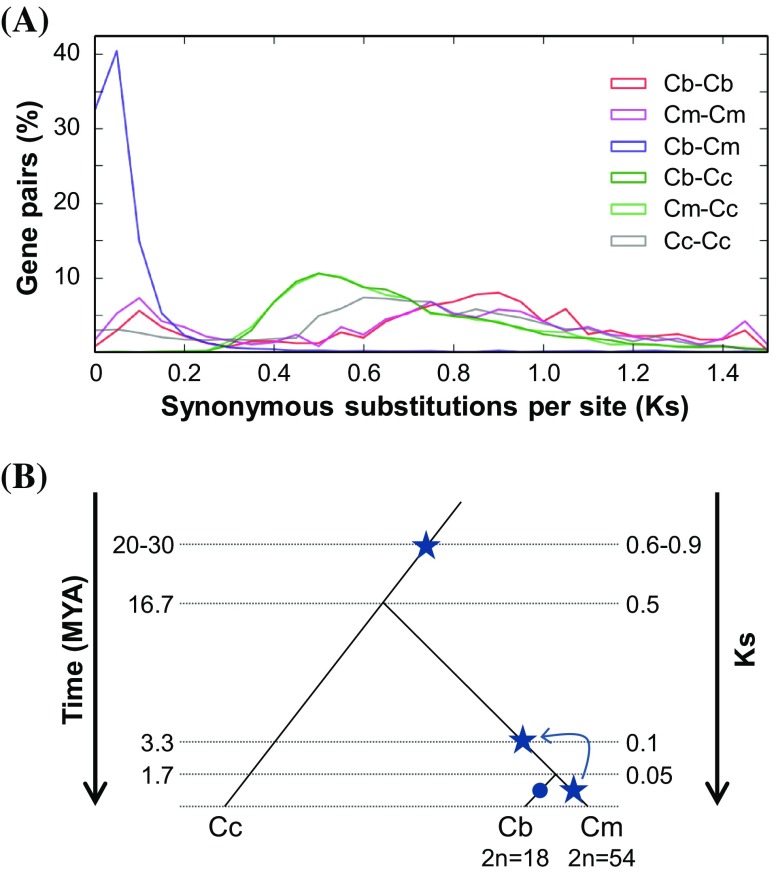
Divergence and whole-genome duplication in *Chrysanthemum*. **a** Distribution of synonymous nucleotide substitutions (Ks) between orthologs and paralogs from *C. boreale* (Cb), *C. morifolium* (Cm), and *Cynara cardunculus* var. *scolymus* (Cc). **b** A proposed model describing the evolutionary events in *Chrysanthemum*. Stars and circle indicate whole-genome duplication (or triplication) and diploidization, respectively. The recent polyploidy is not specific to *C. morifolium* but is instead shared with other members of the *Chrysanthemum* genus

To investigate the divergence time of and WGD in two *Chrysanthemum* species, we examined the distribution of Ks values for orthologous gene pairs and paralogous gene pairs, respectively. At the level of the Asteraceae family, two Ks distributions for orthologs between each *Chrysanthemum* species and *Cynara* (Cb–Cc, Cm–Cc) were perfectly overlapping and showed a peak at Ks = 0.5 (Fig. 4a). Between *C. boreale* and *C. morifolium*, the Ks distribution of orthologous gene pairs (Cb–Cm) showed a prominent peak at 0.05, while a comparison of paralogous gene pairs within each species revealed a relatively sharp Ks peak at 0.1 as well as a broad peak around Ks = 0.9 for *C. boreale* (Cb–Cb) and Ks = 0.75 for *C. morifolium* (Cm–Cm) (Fig. 4a). The paralogous pairs for *Cynara cardunculus* var. *scolymus* (Cc–Cc) also showed a broad peak at Ks = 0.6 (Fig. 4a). Based on the Ks value distributions, *Chrysanthemum* and *Cynara* likely shared an ancient WGD (Ks = 0.6–0.9) and then diverged (Ks = 0.5) (Fig. 4b). Additionally, two *Chrysanthemum* species appear to have experienced a WGD or whole-genome triplication (WGT) event (Ks = 0.1), as well as a species divergence (Ks = 0.05) (Fig. 4b). We estimated the timing of these evolutionary events using the positions of Ks peaks and clock-like synonymous substitution rates for dicots (Koch et al. 2000). We inferred that the Asteraceae species experienced an ancient WGD event 20–30 million years ago (MYA) and that the *Chrysanthemum* genus diverged from *Cynara* 16.7 MYA (Fig. 4b). In addition, the two *Chrysanthemum* species experienced a WGD event 3.3 MYA and then diverged 1.7 MYA (Fig. 4b).

### Detection of sequence divergence

**Fig. 5:**
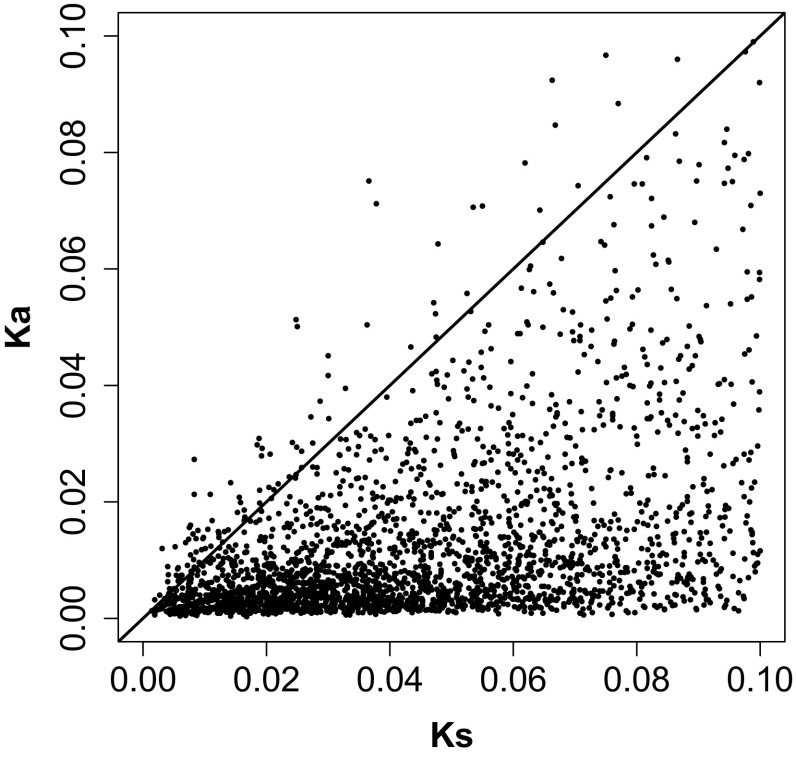
Distribution of non-synonymous (Ka) and synonymous (Ks) nucleotide substitution values of orthologous pairs between *C. boreale* and *C. morifolium*. Orthologous gene pairs with Ka/Ks > 1 are shown above the black solid line

Considering the importance of non-synonymous nucleotide substitutions to protein function and speciation, we also calculated the Ka values and Ka/Ks ratios for orthologs between the two *Chrysanthemum* species. OrthoMCL clustering revealed a total of 4512 orthologous clusters that contained one gene each from *C. boreale* and *C. morifolium*, regardless of the contribution of *Cynara cardunculus* var. *scolymus*. Among these, 137 pairs were identical, with Ka and Ks values of zero, 53 pairs did not result in Ks values, and 118 or 630 pairs had only nonsynonymous or synonymous substitutions, respectively, which were excluded from further analysis. Additionally, 990 orthologous pairs with Ks > 0.1 were known to be potential paralogs and were also removed (Cheng et al. 2015). The remaining 2584 orthologous pairs had both types of substitutions (Fig. 5), although the two species separated very recently. The mean Ka, Ks, and Ka/Ks values were 0.01229, 0.04313, and 0.31642, respectively. We found that 2477 orthologous gene pairs (95.86%) were under purifying selection, with Ka/Ks < 1, whereas 107 pairs (4.14%) were under positive selection, with Ka/Ks > 1 (Fig. 5). Among the genes under positive selection, all except three share sequence similarity with known proteins; these genes are listed in Table S1. These genes encode proteins involved in processes at various levels, such as transcriptional regulation (NAC domain-containing protein, trihelix protein, and CBF1-interacting co-repressor), post-translational modification (heat-shock protein), signal transduction (protein phosphatase 2C-like protein, G protein alpha subunit, GYF-like protein, and TIP41), chromatin condensation (regulator of chromosome condensation repeat), disease resistance (mannose-binding lectin and Kunitz-like protease inhibitor), and so on (Table S1).

## Discussion

In this study, we characterized the transcriptomes of wild and cultivated *Chrysanthemum* species. Sequencing and assembly resulted in the identification of 10,961 and 11,318 genes in *C. boreale* and *C. morifolium*, respectively, with 41,368 and 57,035 unassembled singletons, respectively (Table 1). Given that the number of genes in plant species range from 20,169 to 94,000 (Michael and Jackson 2013), the assembled transcripts represented only a portion of the genes present in each species, but the data obtained in this study were sufficient to allow us to perform evolutionary analysis between the two *Chrysanthemum* species. In addition, GO analysis revealed that the transcriptomes obtained in this study included functionally diverse genes in *Chrysanthemu*m (Fig. 2).

Although the amount of raw data used for transcriptome assembly in both species was similar, the analysis of *C. morifolium* resulted in the discovery of more isogroups, isotigs, and singletons compared to *C. boreale* (Table 1), perhaps due to the differences in genome size and polyploidy. *C. morifolium* is a hexaploid species whose genome size is estimated to be approximately 8.83 Gbp (http://www.kew.org/cvalues), whereas *C. boreale* is a diploid species with a 2.94 Gbp genome (unpublished data, SY Won and J-A Jung). There is a significant positive correlation between the number of genes in an organism and genome size (Hou and Lin 2009). In polyploid species, genome doubling contributes to the expansion and diversification of gene contents, although the subsequent diploidization event involves the loss of a copy of each duplicated gene (Soltis et al. 2015). For example, 35% of genes were lost after the WGT in *Brassica oleracea* compared to the diploid species, *Arabidopsis thaliana* (Town et al. 2006). Indeed, *B. oleracea* was predicted to contain 45,758 genes (Liu et al. 2014), whereas the *A. thaliana* genome contains 25,498 genes (Initiative 2000). In particular, genes involved in the regulation of metabolic and biosynthetic processes and RNA metabolism, as well as transcription factor genes, were over-retained after WGT in *B. oleracea* (Liu et al. 2014).

Comparative genomic and transcriptomic analyses have revealed species- or lineage-specific genes representing major contributors to species- or lineage-specific phenotypes, adaptation, and evolution (Kaessmann 2010). Here, OrthoMCL-based clustering identified gene families that were shared among species or unique to each species (Fig. 3). Since the transcripts obtained in this study represent a subset of genes present in *Chrysanthemum* compared to the genome-based gene set in *Cynara cardunculus* var. *scolymus*, the 2055 *Cynara*-specific gene families were probably overestimated, whereas the 773 *Chrysanthemum*-specific gene families are indeed unique to this genus. In a comparison between *Chrysanthemum* species, we identified genes specific to both the wild and cultivated species, although these genes should be examined again once a more complete gene set is available. Initially, we assumed that genes in diploid species would be triplicated in hexaploid progeny if the two species diverged recently and the extra duplicated gene copies were maintained. Therefore, each orthologous cluster was considered to consist of more genes from *C. morifolium* than from *C. boreale*. Specifically, orthologous groups containing more genes from *C. morifolium* were likely to be more frequent than those from *C. boreale*. However, the clustering results showed the opposite pattern than would be expected with this assumption, perhaps due to WGD and diploidization events in *C. boreale* (see below).

Analysis of the Ks distribution of paralogs revealed two rounds of WGD in *Chrysanthemum* (Fig. 4). According to the timing of the WGD, it appears that the ancient event (Ks = 0.6–0.9) was shared with another Asteraceae member, *Cynara cardunculus* var. *scolymus* (Fig. 4a), whose Ks value overlapped with the Asteraceae-specific Ks peak at approximately 0.7–1.4 (Barker et al. 2016; Huang et al. 2016). Additionally, a peak at Ks = 0.1 was detected (Fig. 4a), which might contribute to the steep background of Ks distribution, revealing the ongoing process of gene birth and death (Blanc and Wolfe 2004). However, it cannot be ruled out that the recent peak is indicative of a hexaploidization event in *C. morifolium*, although it is still unclear whether this event involved autopolyploidy or allopolyploidy. A recent cytological and molecular analysis revealed segmental allohexaploidy in cultivated chrysanthemums (Klie et al. 2014). Notably, *C. boreale* also showed an unusual peak at the same position (Ks = 0.1) (Fig. 4a). Considering the divergence time of the two species at Ks = 0.05, one possible explanation is that a recent WGT event occurred in the common ancestor, and was followed by different evolutionary events in the two species. While *C. morifolium* maintained the original polyploidy status, it is likely that this particular diploid species experienced a rapid diploidization event, including a reduction in chromosome number. This explanation is supported by evidence obtained by OrthoMCL-based clustering. If the recent hexaploidization event occurred only in *C. morifolium* but not in *C. boreale*, orthologous clusters containing more genes from *C. morifolium* are expected to be more frequent. However, the opposite results were obtained, supporting a recent WGD or WGT in *C. boreale*.

Although we found that *C. boreale* and *C. morifolium* diverged from each other very recently, many characteristics of these species are quite different, such as their morphology, resistance, and growth habit, including the observed differences between wild and cultivated species and/or traits that accumulated in *C. morifolium* during the domestication process. One of the most effective approaches for revealing the molecular mechanisms behind these differences is to calculate the Ka/Ks ratios between orthologous gene pairs, especially due to the increasing availability of genomic and transcriptomic sequences. For orthologous gene pairs between the two *Chrysanthemum* species, most (over 95%) are subjected to purifying selection, with Ka/Ks less than one, resulting in the removal of deleterious mutations; this is consistent with the findings for many other plant species (Schlueter et al. 2004). However, the 107 remaining genes are under positive selection, which might influence the adaptation and evolution of each species. We found that these genes are involved in many different aspects of regulation. Among these, one representative class of genes encodes transcription factors or repressors such as NAC (NAM, ATAF1/2, and CUC2) domain-containing protein, trihelix protein, and CBF1 (C-repeat/DRE binding factor 1)-interacting co-repressor. Genes encoding regulator of chromosome condensation repeat (RCC) and Rad21/Rec8-like protein were also found to be under positive selection, conferring an additional layer of transcriptional regulation on the mode of chromosome condensation or structure. In addition, genes involved in signal transduction processes are subjected to evolutionary pressure. These include genes encoding NB-ARC (nucleotide binding adaptor shared by APAF-1, R proteins, and CED-4)-like protein, protein phosphatase 2C-like protein, G protein alpha subunit, glycine-tyrosine-phenylalanine (GYF)-like protein, sensitivity to red light reduced-like (SRR1), and a Tap42-interacting protein (TIP41) in the mTOR signaling pathway.

The most interesting group of genes under positive selection is related to plant defense. Among the genes for signal transduction discussed above are two genes involved in disease resistance. Specifically, ectopic expression of NB-ARC-like protein confers resistance to pathogenic fungi and bacteria in Arabidopsis (Wen et al. 2015), and a protein containing the GYF domain controls the homeostasis of nucleotide-binding leucine-rich repeat (NLR) immune receptors at the level of translational repression in Arabidopsis (Wu et al. 2017). Moreover, other genes related to disease or defense are also under positive selection, including genes encoding mannose-binding lectin (MBL), Kunitz-like protease inhibitor (also known as Kunitz-type trypsin inhibitor, KTI), avrRpt2-induced gene 1 (AIG1), disease resistance protein, cucumber mosaic virus (CMV) 1a interacting protein 1, and tobacco mosaic virus (TMV) resistance protein N-like. MBL was initially reported to be responsible for the recognition of specific carbohydrates on the surfaces of pathogens during infection (Peumans and Van Damme 1995), whereas KTI protects plants by inhibiting the digestive proteases of insects or pathogens (Kim et al. 2009). *AIG1*, which is induced by infection by bacteria carrying the avirulence gene *avrRpt2*, likely elicits differential resistance responses based on the type of avirulence gene and its recognition partner (Reuber and Ausubel 1996). In a comparison of wild versus domesticated plants, genes involved in disease resistance or stress responses were also found to be under positive selection in other Asteraceae species (Kane et al. 2011). Moreover, one outstanding trait found in the wild relatives of domesticated species is better disease and pest resistance compared to their domesticated counterparts (Warschefsky et al. 2014). Indeed, *C. boreale* is more resistant to white rust disease caused by *Puccinia horiana* than *C. morifolium*, (Park et al. 2014). Perhaps stress triggered by pathogens has induced the molecular evolution or positive selection of a broad range of genes for disease resistance or defense during domestication or selection by breeders, which has played an important role in the divergence of the two *Chrysanthemum* species.

Transcriptome sequencing has facilitated the reconstruction of the catalogs of genes in particular species, leading to the identification of genes responsible for specific conditions and helping to track evolutionary events such as WGD, adaptation, and speciation. However, the drawback of RNA sequencing is that the transcripts obtained under-represent the genes in the genome. In addition, the locations of these genes on chromosomes are not identified by this technique. Therefore, even though the present results provide a general overview of WGD, polyploidy, speciation, and adaptation in the *Chrysanthemum* genus, the molecular evolution of the species investigated must be confirmed at the genomic level. First, WGD or WGT could be detected based on the collinear arrangement of genes in the genome, which could exclude the recurrent birth and death of genes from analysis. Therefore, a Ks peak indicating a very recent WGD and subsequent diploidization in *C. boreale* could be obtained without interference from random gene duplication. Second, we expect that more genes involved in the adaptation and speciation could be identified at the genome-wide scale. Indeed, lists of genes annotated as having an unknown function include many genes that experienced rapid evolution for a long period of time and whose identities were consequently obscured; these genes might be excluded from analyses based on the transcriptome (Kane et al. 2011). Similarly, in the current study, as many as 39 positively selected genes (36.4%) were classified as encoding hypothetical, uncharacterized, or unknown proteins or were not functionally annotated. *C. boreale* is currently being subjected to whole-genome sequencing, which is expected to provide a fundamental resource that can be used to accurately reveal the molecular and genomic evolution of the *Chrysanthemum* genus.

## Conclusions

Speciation in *Chrysanthemum* is associated with hybridization, polyploidization, and adaptation. To reconstruct these evolutionary events and to deduce their time of occurrence, we characterized the transcriptomes of a cultivated *Chrysanthemum* species, *C. morifolium*, and its wild relative, *C. boreale*. These analyses not only confirmed the ancient WGD event shared among members of the Asteraceae family, but they also provided evidence for a recent WGD or WGT event specific to *Chrysanthemum*. A comparison of the transcriptomes of these species revealed genes that likely experienced positive selection during the processes of domestication and adaptation, which would be responsible for the divergence of the two species. Among the various conditions, biotic stresses likely play important roles in selection and adaption. These transcriptome-based molecular evolutionary analyses of genomes and genes will be addressed using whole-genome sequences in the future.

## Electronic supplementary material

Below is the link to the electronic supplementary material.

Supplementary material 1 (XLSX 15 KB)
